# RNA-seq of eight different poplar clones reveals conserved up-regulation of gene expression in response to insect herbivory

**DOI:** 10.1186/s12864-019-6048-8

**Published:** 2019-08-28

**Authors:** Niels A. Müller, Birgit Kersten, Matthias Fladung, Hilke Schroeder

**Affiliations:** Thünen Institute of Forest Genetics, Sieker Landstraße 2, 22927 Grosshansdorf, Germany

**Keywords:** *Populus*, Insect herbivory, Co-expression network, WRKY transcription factors, Jasmonate (JA) signaling, G-box, *Phratora vitellinae*

## Abstract

**Background:**

Herbivorous insects can have a profound impact on plant growth performance. In some years, canopy damage in poplar plantations exceeds 50% of the total leaf surface, thereby possibly compromising carbon fixation and biomass yield. To assess the transcriptional response of elite poplar clones to insect feeding and to test whether this response varies between different genotypes, we performed an RNA-sequencing experiment. We deeply sequenced the transcriptomes of eight elite clones belonging to three poplar species (*Populus trichocarpa*, *P. nigra* and *P. maximowiczii*), under *Phratora vitellinae* feeding and control conditions. This allowed us to precisely quantify transcript levels of about 24,000 expressed genes.

**Results:**

Our data reveal a striking overall up-regulation of gene expression under insect attack in all eight poplar clones studied. The up-regulated genes were markedly enriched for the biological process ‘regulation of transcription’ indicating a highly concerted restructuring of the transcriptome. A search for potential cis-regulatory elements (CREs) that may be involved in this process identified the G-box (CACGTG) as the most significant motif in the promoters of the induced genes. In line with the role of the G-box in jasmonate (JA)-mediated activation of gene expression by MYC2, several genes involved in JA biosynthesis and signaling were up-regulated in our dataset. A co-expression network analysis additionally highlighted WRKY transcription factors. Within the most prominent expression module, WRKYs were strongly overrepresented and occupied several network hubs. Finally, the insect-induced genes comprised several protein families known to be involved in plant defenses, e.g. cytochrome P450s, chitinases and protease inhibitors.

**Conclusions:**

Our data represent a comprehensive characterization of the transcriptional response of selected elite poplar clones to insect herbivory. Our results suggest that the concerted up-regulation of gene expression is controlled by JA signaling and WRKY transcription factors, and activates several defense mechanisms. Our data highlight potential targets of selection and may thus contribute to breeding insect-resistant poplar clones.

**Electronic supplementary material:**

The online version of this article (10.1186/s12864-019-6048-8) contains supplementary material, which is available to authorized users.

## Background

Plants evolved highly sophisticated and strictly regulated defense mechanisms to fight off diseases and pests, e.g. herbivorous insects [1]. Defense responses include direct (repellents) and indirect mechanisms (attracting of parasitoids and predators) [2]. For their direct defense, plants are using a reservoir of more than 200,000 secondary metabolites [3, 4]. Several of these metabolites are constitutively expressed, thereby ensuring a constant level of basic protection [1, 5]. Another part is activated only when plants are being attacked, completing the plants’ armament against herbivores [6, 7]. This highly regulated response is crucial for minimizing the trade-offs between growth and defense – the metabolic pathways important for reducing damage caused by pests and diseases must be balanced with the biological processes optimizing vegetative growth [8–10].

A key compound for the regulation of the plant’s defense against herbivorous insects is JA [11]. It has been first connected to the defense response about 30 years ago [12] and has since then been investigated intensively [13–15]. The relevance of its proper diurnal timing again points to the importance of precise transcriptional regulation to minimize trade-offs [16]. JA leads to global reprogramming of gene expression, activating various toxic secondary metabolites and defensive proteins [11]. Among the defensive proteins, protease inhibitors play a prominent role. They reduce the plants’ nutritional value by negatively influencing the digestion capacity of the insects [17]. Other examples of plant defensive proteins, many of which are tightly regulated by the JA signaling pathway, include lectins and chitinases [11]. Plant chitinases directly antagonize insects by destroying chitin, a major component of the insect’s exoskeleton [18]. Finally, the induced release of plant volatiles can mediate direct or indirect defense against herbivorous insects [19, 20].

Plant-pathogen and plant-herbivore interactions are characterized by reciprocal selection pressures that lead to a continuous arms race [21, 22]. Trees have an inherent disadvantage in this evolutionary process due to their often very long generation times that prevent rapid adaptation. Tree species thus require defense mechanisms that remain effective throughout their long life cycle. They basically need to be pre-adapted to evolving pests and diseases, for example by possessing a high diversity of innate defense strategies. In fact, tree genomes in general show an amplification of defense genes [23], and also the poplar genome in particular is enriched for genes associated with disease and insect resistance [24].

Poplars are economically important tree species in Europe and North America because of their high growth rate and their broad applicability ranging from wood and paper to energy production [25]. Several poplar species are cross-compatible leading to a high number of artificially and naturally produced interspecific hybrids [26]. Since many of these crosses exhibit hybrid vigor, they are commonly used in short rotation plantations for biomass production. Additionally, the genus *Populus* represents a model species for tree genetics and genomics, due to its small genome size, and huge genomic and biotechnological resources. The genome of western black cottonwood, *P. trichocarpa*, was the third plant genome to be published, only after *Arabidopsis* and rice [24]. Although previous analyses of poplar gene expression in response to insect feeding provided valuable insights [27–29], an RNA-sequencing analysis across different *Populus* species has, to our knowledge, not been performed. The relevance of the different defense mechanisms against herbivorous insects thus remains to be determined in this important tree genus.

In the present work, we aimed to identify the genes and molecular pathways shaping the defense response of different poplar species against herbivorous insects. To this end, we applied RNA-sequencing to precisely characterize the transcriptomes of eight elite poplar clones belonging to three different species under insect feeding and control conditions. We used well-characterized clones from the ‘FastWOOD’ project [30, 31] in order to facilitate transferability of our results into ongoing breeding efforts aiming at the development of less susceptible highly yielding poplar clones.

## Results

### The response of poplars to insect herbivory is characterized by conserved up-regulation of gene expression

**Fig. 1:**
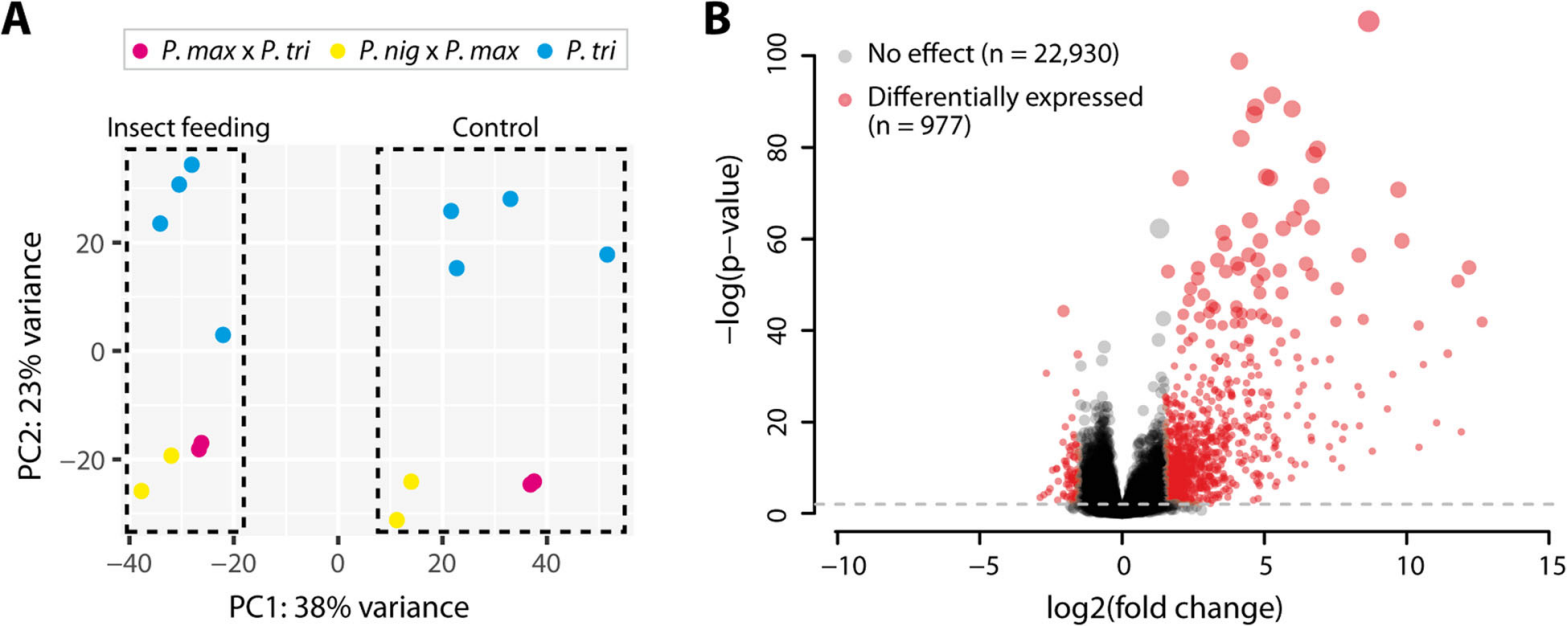
Insect feeding causes conserved up-regulation of gene expression across eight elite poplar clones. **a** Principal component analysis (PCA) shows clustering of RNA-seq samples by treatment and species. While PC1, explaining 38% of the total variance, separates treated samples from control samples, PC2, explaining 23% of the total variance, differentiates pure P. trichocarpa clones (blue) from the hybrids P. maximowiczii x P. trichocarpa (red) and P. nigra x P. maximowiczii (yellow). **b** Volcano plot showing the 23,907 expressed genes. Significantly differentially expressed genes in response to insect feeding (adjusted *P* < 0.01 and absolute log2 fold change > 1.5) are depicted in red. While 885 genes are up-regulated, only 92 genes exhibit down-regulation under herbivory. The size of the dots corresponds to the *p*-value. The dashed line indicates the p-value significance threshold

To characterize the transcriptional response of poplars to herbivorous insect attack and to identify possible differences between genotypes, we sequenced the RNA of eight elite poplar clones under insect feeding and control conditions. This yielded an average of about 60 million mapped reads per clone and condition using the *P. trichocarpa* reference genome v3.0 [24] (Additional file 1: Table S1). We first analyzed the overall variation of the samples in our dataset with a principle component analysis (PCA). This PCA demonstrated that most of the variation in the transcriptomes is caused by the treatment (insect feeding vs. control conditions) (Fig. 1 a).

Additional differences between samples are due to genotype. Especially pure *P. trichocarpa* clones differed from the hybrid clones (*P. maximowiczii* x *P. trichocarpa* and *P. nigra* x *P. maximowiczii*) (Fig. 1 a). However, there was no significant interaction between the response to herbivore attack and genotype (*P* > 0.01 for ~treatment*clone or ~treatment*species). We therefore analyzed all samples together for assessing the effect of herbivorous insect attack on the poplar transcriptome. It must be noted, however, that the lack of replication within each genotype limits the statistical power to identify clone-specific effects. The comparison between the four pure *P. trichocarpa* clones and the four hybrid clones, on the other hand, should be statistically robust, indicating that there is no differential response due to taxonomy. The joint differential expression analysis identified 977 genes that respond to insect feeding. Interestingly, the large majority of these, i.e. Eight hundred eighty-five genes, are up-regulated compared to only 92 down-regulated genes (Fig. 1 b and Additional file 1: Table S2). Although visualization of the top 50 up- and down-regulated genes showed notable variability between genotypes (Additional file 2: Figure S1), the PCA and the differential expression analysis demonstrate that the response of poplars to herbivorous insects is largely conserved across genotypes and characterized by a pronounced up-regulation of gene expression (Fig. 1).

### WRKY transcription factors and JA signaling are important for concerting the transcriptional response

**Fig. 2:**
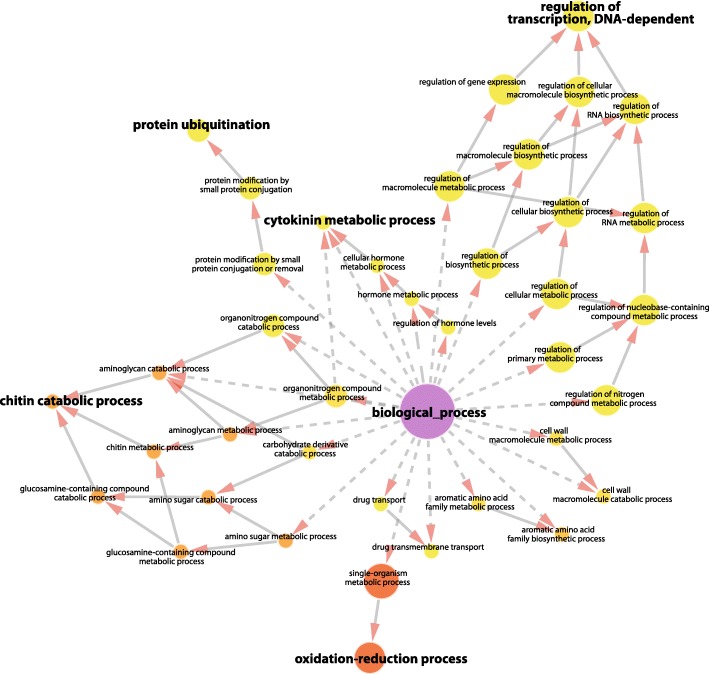
Up-regulated genes are especially enriched for regulators of DNA-dependent transcription. GO term analysis particularly highlighted the biological processes ‘regulation of transcription’, ‘chitin catabolic process’, ‘oxidation-reduction process’, ‘protein ubiquitination’ and ‘cytokinin metabolic process’, indicated by bold font. The color of the circles shows the significance level of each GO term (from yellow = *P* < 0.05 to red = *P* < 10–7), their size indicates the number of comprised differentially expressed genes

In order to better understand what kind of genes or pathways are up-regulated in response to herbivore attack, we performed a gene ontology (GO) term enrichment analysis. This analysis identified several GO terms that are overrepresented among the up-regulated genes (Fig. 2). Among the enriched categories, one of the most prominent biological processes was ‘regulation of transcription’ (Fig. 2). This highlights the importance of transcription factors in reorganizing the transcriptome of poplars attacked by insects and points to a highly concerted transcriptional response.

Although only 10% of the differentially expressed genes were down-regulated by insect herbivory, enrichment of functional categories was found among those genes as well (Additional file 2: Figure S2). However, the biological relevance of the functional categories (‘proteolysis’ and ‘carotenoid biosynthetic process’) enriched among the down-regulated genes was less obvious than for the up-regulated genes.

In case of a concerted transcriptional response, as indicated by the enrichment of transcription factors among the up-regulated genes, differentially expressed genes may share common cis-regulatory elements (CREs) [32]. To test whether the promoters of the herbivore-induced genes comprise any specific 6–10 bp sequence motifs compared to randomly sampled non-differentially expressed genes, we performed a differential motif enrichment analysis using the MEME tool [33]. Employing different reference sets of randomly sampled genes, we consistently identified the G-box (CACGTG) as the most significantly enriched motif (Additional file 2: Figure S3). The G-box is known to be bound by bHLH and bZIP transcription factors [34]. One such bHLH transcription factor is MYC2, also known as JASMONATE-INSENSITIVE 1 [35]. MYC2, which is significantly up-regulated by insect feeding in our dataset (log2FC = 1.7; Additional file 1: Table S2, Potri.003G092200), is a master regulator of JA induced gene activation [36, 37]. JA signaling in turn has a firmly established role in the induced defense against insect herbivores [11, 15]. Remarkably, a closer examination of the enriched functional categories of the herbivore-induced genes in our data revealed several groups involved in JA signaling and biosynthesis. Namely, the jasmonate ZIM domain-containing (JAZ) proteins, which are early targets of JA-induced gene expression [15], represent the most significantly enriched KEGG orthology (KO) term (Table 1). Additionally, the KOs ‘lipoxygenase’ and ‘allene oxide cyclase’, which both comprise important genes of the JA biosynthesis pathway (e.g. LOX and AOC genes), were among the top 5 enriched terms. Finally, further genes involved in JA biosynthesis, i.e. *OPR3* (Potri.018G065600) and *ACX1* (Potri.001G155500) [15], were significantly up-regulated as well (log2FC = 6.0 and 2.4, respectively; Additional file 1: Table S2). Together these data establish a distinctive role of JA signaling in response to insect feeding in poplar and indicate an involvement of the G-box, potentially by guiding the binding of MYC2.

**Table 1:** Top 5 most significantly enriched KEGG orthology (KO) annotations among the herbivore-induced genes

KO	*P*-value	Contingency table		Description
K13464	2.16E-06	5/150	6/5582	jasmonate ZIM domain-containing protein
K00454	1.20E-05	6/150	14/55821	lipoxygenase
K08235	3.69E-04	6/150	25/5582	xyloglucan:xyloglucosyl transferase
K13993	3.98E-04	4/150	9/5582	HSP20 family protein
K10525	2.87E-03	2/150	2/5582	allene oxide cyclase

**Fig. 3:**
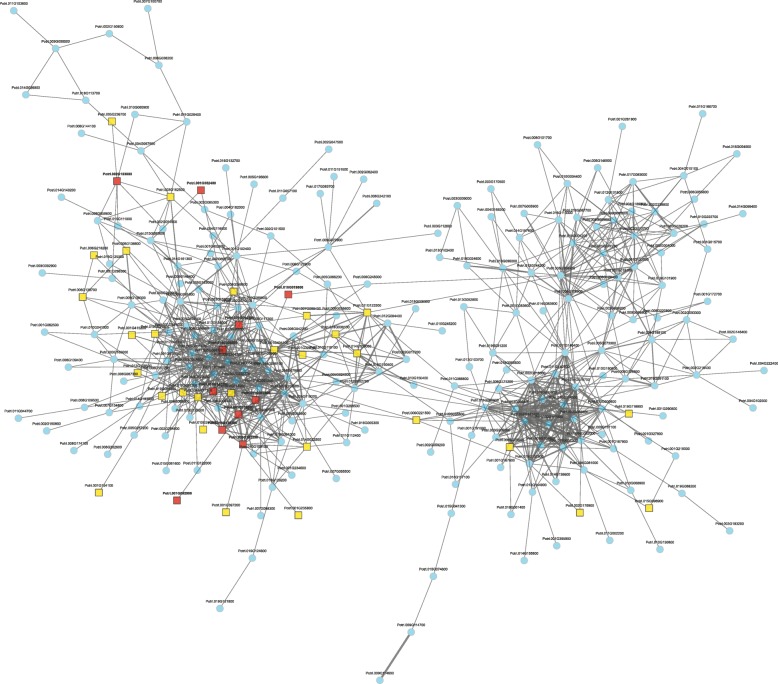
Co-expression network highlights WRKY transcription factors. From the 885 up-regulated genes, about 30% (258) formed a two-clustered co-expression module. Co-expressed genes are connected by lines, whose widths correspond to the strength of the interaction. Transcription factors (depicted in yellow) and particularly WRKYs (shown in red) are significantly overrepresented (adjusted *P* = 2.92E 08, Additional file 1: Table S4)

Concerted reorganization of the transcriptome is not only expected to involve shared regulatory sequence motifs, but also co-expressed gene networks. Network hubs should highlight important regulators of the transcriptional response [38]. To explore this possibility and to assess which genes may be crucial for herbivore-induced gene expression, we performed a co-expression network analysis. From the 885 herbivore-induced genes, about 30% exhibited co-expression across several hundred microarrays [39] (Additional file 2: Figure S4 and Additional file 1: Table S3). This network formed two separate expression modules (Fig. 3). Notably, the more prominent of those two modules was significantly enriched for WRKY transcription factors (Additional file 1: Table S4, *P* = 2.92E-08) and several of those WRKYs represented network hubs (Fig. 3). In addition to JA signaling, WRKY transcription factors thus appear to play an important role in regulating the restructuring of the transcriptome in response to herbivore attack in poplars.

### Cytochrome P450s, chitinases and protease inhibitors are induced by insect herbivory

To further explore which genes or processes may be activated by MYC2, WRKYs and additional transcription factors, we performed a final close examination of the herbivore-induced genes. Besides the established importance of transcriptional regulation (namely by AP2 domain and WRKY domain containing transcription factors) and JA signaling (represented by the tify domain containing JAZ proteins), we found many cytochrome P450 proteins (Table 2). In plants, cytochrome P450s are involved in diverse biosynthetic processes including the biosynthesis of defense compounds that can be involved in the induced direct defense against herbivores [40, 41]. Another highly enriched group of genes comprises the plant-specific class I chitinases (Table 2). They likely represent a direct defense against the insects as they can destroy their chitin-containing exoskeleton. Finally, trypsin and protease inhibitors are markedly enriched among the herbivore-induced genes (Table 2). These proteins are known to have an anti-nutritional effect by hindering digestion, and thus likely also serve as a direct defense against the insects by compromising the nutritional value of the attacked poplar leaves. In conclusion, among the herbivore-induced genes we find several enriched protein families that appear to be concerting and carrying out the defense against the attacking insects.

**Table 2:** Most significantly enriched PFAM categories among herbivore-induced genes

PFAM/KO	*P*-value	Contingency table	Description
PF06200	4.39E-10	10/788 | 17/29014	tify domain
PF00067	5.49E-09	39/788 | 421/29014	Cytochrome P450
PF00847	7.87E-09	27/788 | 209/29014	AP2 domain
PF00182	1.38E-06	8/788 | 21/29014	Chitinase class I
PF00197	2.67E-06	9/788 | 32/29014	Trypsin and protease inhibitor
PF03106	2.86E-06	15/788 | 102/29014	WRKY DNA -binding domain

## Discussion

In this paper, we describe a deep RNA sequencing dataset of eight elite poplar clones belonging to three different species. Overall, we found marked differences between samples due to condition (insect feeding vs. control) and genotype. Especially the pure *P. trichocarpa* clones differed from the hybrid clones, i.e. *P. nigra* x *P. maximowiczii* and *P. maximowiczii* x *P. trichocarpa*. Interestingly though, there was no significant interaction between the response to herbivorous insects and taxonomy. Although the detection of particular differences of single clones is limited due to lack of replication, the results indicate a largely conserved transcriptional response. This response is characterized by a pronounced up-regulation of gene expression. Only a small minority of the differentially expressed genes are down-regulated (92 vs. 885). Nevertheless, this down-regulation may be biologically relevant. For example, genes involved in carotenoid biosynthesis are enriched among the down-regulated genes in our dataset. Most animals, including crysomelid beetles, rely on their diet for obtaining carotenoids. Interestingly, some aphids can endogenously synthesize carotenoids, which likely contribute to a healthy immune system, interactions with natural enemies and even sunlight-harvesting [42–44]. The down-regulation of carotenoid biosynthesis in poplars may reduce the availability of these essential nutrients and thus compromise herbivore performance.

Whether genes are up- or down-regulated in response to a stress factor can depend on the tissue, the genotype, the time-point and the treatment. Simulated feeding experiments using *Malacosoma disstria* oral secret, for example, led to a higher amount of up- than down-regulated genes in fully developed leaves of *P. trichocarpa* x *P. deltoides* [28, 29]. This is in concordance with our study, which also used fully developed leaves for the feeding experiments. The temporal pattern of the transcriptional response revealed more pronounced effects at 2 h and 6 h after the treatment, compared to 24 h [29]. In contrast, we still found a large number of up-regulated genes after 21 h of insect feeding. It would be interesting to perform an RNA-seq time-course including very early time-points, to more precisely define the early transcriptional dynamics in response to insect herbivory. This may also allow disentangling primary from secondary responses, which could help to select targets of selection for poplar breeders.

In line with the pronounced up-regulation of gene expression, we identified many transcription factors among the herbivory-induced genes and ‘regulation of transcription’ as one of the most prominently enriched GO terms. Especially WRKY transcription factors appeared to play an important role in orchestrating the transcriptional response, as they occupied important positions in a co-expression network of the induced genes. The involvement of WRKYs in the response to abiotic (salt stress) as well as biotic factors (pathogenic fungi and even herbivorous insects) has been previously shown in poplars [45–47]. Our data add additional emphasis on the role of WRKY transcription factors for regulating the highly conserved transcriptional response to insect feeding. Especially the herbivory-induced WRKYs representing hub genes in our co-expression network (i.e. Potri.001G044500: WRKY40, Potri.003G138600: WRKY53, Potri.003G182200: WRKY40, Potri.010G147700: WRKY48, Potri.011G007800: WRKY42, Potri.016G128300: WRKY33 and Potri.018G008500: WRKY11) may serve as master regulators. Further functional analyses of these specific genes in poplar, for example via overexpression or CRISPR/Cas-mediated knockout, could yield interesting insights into their individual contributions to the induced defense against insect pests.

Another crucial pathway for the restructuring of the transcriptome in response to herbivorous insects is the JA pathway. It is rapidly induced but also tightly regulated. A negative feedback loop, represented by the JASMONATE ZIM-domain (JAZ) transcriptional repressors, is simultaneously up-regulated [48]. Accordingly, besides genes involved in JA biosynthesis and signaling, we found many JAZ genes among the insect-induced genes. The canonical JA signaling pathway thus appears to be activated in poplars challenged by insect feeding. Interestingly, the G-box, which is a cis-regulatory element (CRE) recognized by the master regulator of JA signaling MYC2, is the most significantly enriched DNA motif in the promoters of the insect-induced genes. It may contribute to the regulation of transcription factors and enzymes around the JA pathway.

The binding of MYC transcription factors to the G-box has also been shown for two very distinct tree species, *Taxus* sp. and *Hevea brasiliensis* [49, 50]*.* The more general role of the G-box, however, remains to be characterized in trees. In *Arabidopsis*, on the other hand, the G-box has been intensively investigated. Even though the prediction of regulatory relationships is difficult, because many bHLH and bZIP transcription factors bind to the G-box, a gene expression network revealed likely bHLH and bZIP regulatory targets [34]. This network accurately reconstructed known subnetworks and was able to predict transcriptional patterns [34]. A similar analysis of the G-box-associated transcription factors and their potential downstream targets in poplars may yield interesting insights into the regulation of important biological processes in a tree species.

Defensive proteins induced by JA signaling include plant chitinases. Genes encoding plant chitinases were among the first genes identified to respond to insect feeding in poplars [51]. They represent an effective direct defense as they destroy the exoskeleton of insects. Thus, it is not surprising that chitinase genes were highly enriched in our dataset. Our data highlight 8 of the 21 genes with the PFAM annotation ‘chitinase class I’. Among those, and also among the most strongly differentially expressed genes in general (Additional file 1: Table S5), are three poplar homologs of the *Arabidopsis PATHOGENESIS-RELATED 3* (*PR3*) gene (Potri.004G182100, Potri.009G141800 and Potri.009G142000; log2FC = 9.7, 8.3 and 7.5, respectively), one of which has been demonstrated to confer insect resistance across species [52]. These poplar genes thus represent prominent targets for resistance breeding.

An ever increasing demand for silvicultural production is putting high pressure on modern forestry. Changing environmental conditions and newly introduced pests and diseases, including insects, pose additional challenges. Since biotechnological solutions involving genetic engineering do not find consumers’ acceptance in Europe, alternative ways are needed to enhance forest tree production in a relatively short timeframe. Tree breeding can be accelerated by integrating knowledge of the genes and gene networks underlying target traits. Although the effects of mycorrhizal fungi or rhizosphere microbes can outweigh host plant genetics in reducing insect herbivory [53, 54], natural genetic variation still provides a great potential. The comprehensive characterization of the poplar transcriptome in response to insect herbivory, described here, may serve as a starting point for identifying natural gene variants, which may match the success of conferring enhanced resistance by transgenic approaches [55]. Although functional genomics datasets don’t provide simple directions for breeding programs, they do generate a wealth of information that can be integrated with other data, or inform the design of follow-up experiments, to support trait improvement. QTL or GWA studies, for example, can greatly benefit from integrating RNA-seq data for the identification of the causal gene variants [56, 57], which can then be used for marker-assisted selection. Furthermore, reverse genetics experiments can be designed based on the results of transcriptomics studies. Artificial deregulation of promising candidate genes can give crucial insights into their individual contribution to the trait of interest. As trade-offs between growth and defense play an important role, pleiotropic effects need to be given special attention. Along these lines, genes encoding specific defensive proteins may be worth further exploring. On the other hand, changing the activity of a single transcription factor can potentially amplify the desired effect, albeit with a higher risk of pleiotropy. For poplar breeding we believe that several routes should be pursued in parallel. Genetic mapping studies can highlight genomic regions important for naturally occurring enhanced resistance. Functional analysis of specific candidate genes may additionally highlight ways of improving insect resistance that are not commonly used by nature. In any case, the variety of available resources and methods offers exciting possibilities for the future.

## Conclusions

In conclusion, we present a comprehensive characterization of the poplar transcriptome in response to insect herbivory. We identify herbivory-induced genes and signaling pathways widely conserved across different plant species, e.g. the JA signaling pathway, including MYC2, WRKY transcription factors and plant chitinases. Additionally, our data specifically highlight poplar genes potentially representing important regulators of the induced defense against insect herbivores. Further work on those genes may yield valuable insights into their functional relevance and natural variability, and may thereby contribute to poplar breeding.

## Methods

### Plant material and growth conditions

Eight poplar clones were chosen for RNA sequencing out of 20 clones used in the FastWOOD project [31]. Six clones are well established and already used for decades in short rotation plantations, two are newly bred by the “Nord-Westdeutsche Forstliche Versuchsanstalt” (NW-FVA, Hann. Münden, Germany) within the FastWOOD project (Table 3) [31].

**Table 3:** Poplar clones used in this study

Clone name	Species	Status
Rochester	*P. nigra* x *P. maximowiczii*	Established
Max1	*P. nigra* x *P. maximowiczii*	Established
Androscoggin	*P. maximowiczii* x *P. trichocarpa*	Established
NW7_197S	*P. maximowiczii* x *P. trichocarpa*	Newly bred [31]
Weser4	*P. trichocarpa*	Established
Weser6	*P. trichocarpa*	Established
Muhle-Larsen	*P. trichocarpa*	Established
NW7_17C	*P. trichocarpa*	Newly bred [31]

In April and May 2015, branches from the eight clones were harvested at different experimental sites and brought into tissue culture to synchronize their development. In January 2016, 55 to 60 head cuttings of each clone were rooted and transferred from tissue culture into soil. After 2 weeks in controlled environmental chambers, the plants were transferred to the greenhouse. In the following weeks, all plants were transferred to larger pots two times. In May 2016, all plants were cut to a length of 35 cm, covered with gauze to prevent insect attack, and transferred into an open but slightly shaded hall for toughening of the plants. All plants were exposed to the same experimental and environmental conditions. Fertilizer was applied once in spring 2016.

### Feeding experiment

In June 2016, 20 healthy plants of nearly the same size of each clone were chosen for the feeding experiment. Five individuals of the brassy willow beetles (*Phratora vitellinae*) were placed on each of 10 plants of each clone. Next to the plants with insects the same number of control plants without insects were placed also covered with gauze. Thus, in total for each clone 20 ramets were used. After 21 h, the insects were removed from the plants, three leaves from every damaged and control plant were harvested and immediately transferred into liquid nitrogen for later RNA extraction. Thus, we had 10 biological replicates per clone and treatment and three technical replicates per ramet.

### RNA-extraction and sequencing

Total RNA from all 480 samples from the feeding experiment was extracted individually by applying the innuPREP Plant RNA Kit (Analytik Jena AG, Jena, Germany). To avoid DNA contamination, the Invitrogen TM Ambion Turbo DNA free Kit (Fisher Scientific GmbH, Schwerte, Germany) was used following the manufacturer’s instructions.

Quantity of the RNA was determined with a Nanodrop 1000 spectrophotometer (Thermo Fisher Scientific, Wilmington, USA). The quality was measured with the Bioanalyzer Agilent 2100 (Agilent Technologies, Waldbronn, Germany). For each clone, the five samples from five different plants (biological replicates) with the best quality values were pooled. For each of the 16 pools, strand-specific cDNA libraries were created from 1 μg of RNA (GATC Biotech AG, Konstanz, Germany). All libraries were sequenced by GATC Biotech AG (Konstanz, Germany) on an Illumina HiSeq 2500 platform to create 125 bp paired-end reads (on average 70 million reads per sample, Additional file 1: Table S1).

### RNA-seq data analysis

Fastq files were trimmed and filtered using Trimmomatic v0.35 [58] with the following parameters: ILLUMINACLIP: <fastaWithAdapters>:2:30:10 LEADING:3 TRAILING:3 SLIDINGWINDOW:4:20 MINLEN:50. Specific adapter sequences are given in Additional file 1: Table S6. We mapped and quantified the trimmed and filtered reads according to the *Populus trichocarpa* v3.0 reference genome [24] using the STAR aligner [59] with the following parameters: --outSAMtype BAM SortedByCoordinate --quantMode GeneCounts --outReadsUnmapped Fastx --alignIntronMax 11,000. For read quantification per transcript we converted the gff3 file to gtf using the gffread utility included with the Cufflinks package [60]. For subsequent analyses, we only considered genes with rpkm (reads per kilo base per million mapped reads) values > 1 for at least 50% of all samples. Twenty-three thousand nine hundred seven genes passed this filtering step. Finally, we employed the R bioconductor’s package DESeq2 [61] to normalize the read counts using the rlog function and to determine differentially expressed genes (design = ~ Treatment+Clone). Differentially expressed genes were defined as having an adjusted *p*-value < 0.01 and an absolute log2 fold change > 1.5.

### Cis-regulatory element analysis

The putative promoters of differentially regulated genes, i.e. One kb upstream sequence of the transcriptional start site, were analyzed for enrichment of potential cis-regulatory elements (CREs) using the MEME tool within the online MEME Suite 5.0.2. (meme-suite.org/tools/meme) [33, 62]. To compare the promoter sequences of the 885 up-regulated genes with 885 randomly sampled non-differentially expressed genes, we used the differential enrichment mode. We searched for motifs between 6 and 10 bp with any number of repetitions, employing three different sets of randomly sampled genes. Only motifs found in all three comparisons were considered significant.

### Enrichment and co-expression network analyses

GO term, KEGG orthology (KO) and PFAM enrichment analyses as well as co-expression network calculations were performed online using the web-tools available on popgenie.org [39]. For the enrichment analyses we only considered significant terms (*P* < 0.05) with at least 2 genes. For the co-expression network analysis we used the ‘All Affymetrics’ dataset, comprising expression data from 462 Affymetrix microarray samples, and the CLR (context likelihood of relatedness) correlation with default parameters. All other data analyses and visualization were performed using R [63].

## Additional files

Additional file 1: **Table S1.** Number of RNA-seq reads and percentage of uniquely mapped reads. **Table S2.** Differential expression results from DESeq2. **Table S3.** Genes forming the co-expression module shown in Fig. 3. **Table S4.** PFAM enrichment analysis of the genes forming the co-expression module shown in Fig. 3. **Table S5.** Most strongly induced genes, defined as having top 10% adjusted *p*-values and log2 fold-changes. Data for the genes were extracted via the GeneList utility on popgenie.org. **Table S6.** Illumina adapter sequences for each RNA-seq sample. (XLSX 2557 kb)

Additional file 2: **Figure S1.** Heatmaps presenting scaled rpkm-values of the top 50 up- and down-regulated genes. Plots were produced with the heatmap() fuction in R with default settings. Pure *P. trichocarpa* samples are indicated by blue font. **Figure S2.** GO term analysis of down-regulated genes highlighted the biological processes ‘proteolysis’ and ‘carotenoid biosynthetic process’. **Figure S3.** Differential motif enrichment analysis by MEME consistently identified the G-box (CACGTG) as the most significant motif. Promoters (1 kb sequence upstream from the transcriptional start site) of the 885 herbivore-induced genes were compared to those of three randomly sampled sets (A to C) of non-induced genes. **Figure S4.** Co-expression network analysis revealed a network comprising 258 out of the 885 herbivore-induced genes. The network forms two expression modules. Co-expressed genes are connected by lines, whose widths correspond to the strength of the interaction. Transcription factors are depicted in yellow. (PDF 3830 kb)

## Data Availability

All RNA-seq data have been deposited at SRA (NCBI) with the accession codes PRJNA514029 for the clones ‘Muhle Larsen’, ‘NW7_17C’, ‘Weser4’ and ‘Weser6’ [64] and PRJNA523796 for the clones ‘Max1’, ‘Androscoggin’, ‘Rochester’ and ‘NW7_197S’.

## Abbreviations

CLR: Context likelihood of relatedness
CRE: Cis-regulatory element
GO: Gene ontology
JA: Jasmonate
JAZ: Jasmonate ZIM domain
KO: KEGG orthology
PCA: Principle component analysis
PR3: PATHOGENESIS-RELATED 3
