# Supplementary material for: RNA-seq of eight different poplar clones reveals conserved up-regulation of gene expression in response to insect herbivory
Source: BMC Genomics. 2019 Aug 28;20:673. doi: 10.1186/s12864-019-6048-8 (PMC6712675; doi:10.1186/s12864-019-6048-8)

Fig. S1

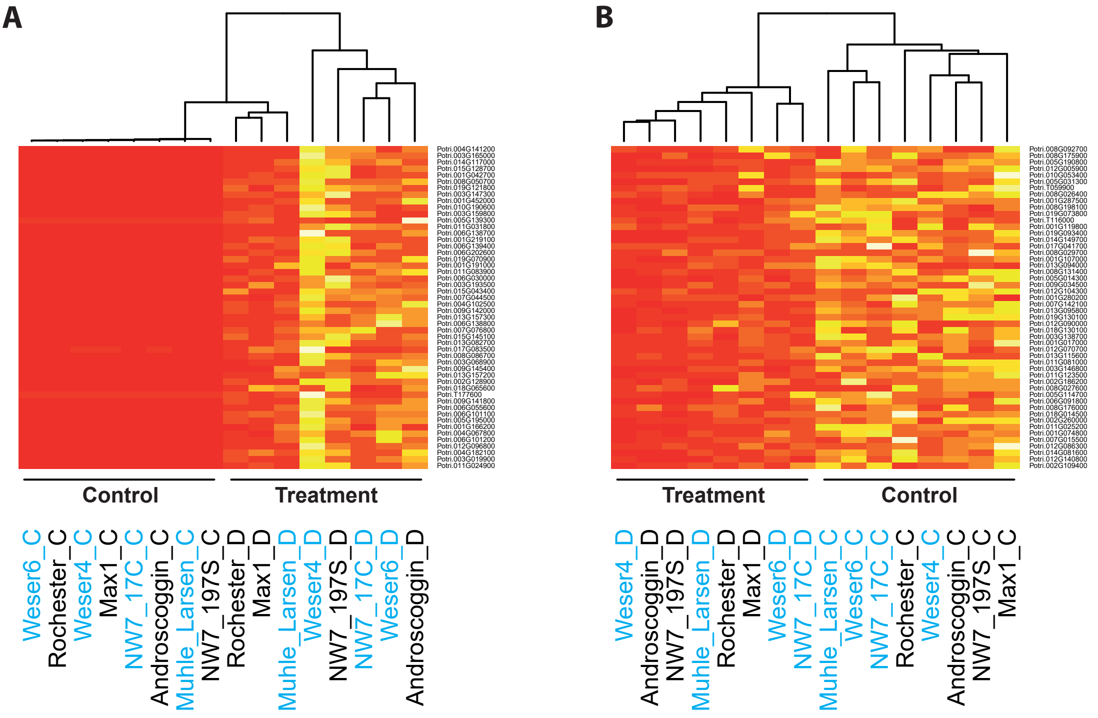

A
Potri.004G141200
Potri.003G165000
Potri.014G117000
Potri.015G128700
Potri.001G042700
Potri.008G050700
Potri.019G121800
Potri.003G147300
Potri.001G452000
Potri.010G190600
Potri.003G159800
Potri.005G139300
Potri.011G031800
Potri.006G138700
Potri.001G219100
Potri.006G139400
Potri.006G202600
Potri.019G070900
Potri.001G191000
Potri.011G083900
Potri.006G030000
Potri.003G193500
Potri.015G043400
Potri.007G044500
Potri.004G102500
Potri.009G142000
Potri.013G157300
Potri.006G138800
Potri.007G076800
Potri.015G145100
Potri.013G082700
Potri.017G083500
Potri.008G086700
Potri.003G068900
Potri.009G145400
Potri.013G157200
Potri.002G128900
Potri.018G065600
Potri.T177600
Potri.009G141800
Potri.006G055600
Potri.006G101100
Potri.005G195000
Potri.001G166200
Potri.004G067800
Potri.006G101200
Potri.012G096800
Potri.004G182100
Potri.003G019900
Potri.011G024900
Control
Treatment
Weser6_C
Rochester_C
Weser4_C
Max1_C
NW7_17C_C
Androscoggin_C
Muhle_Larsen_C
NW7_197S_C
Rochester_D
Max1_D
Muhle_Larsen_D
Weser4_D
NW7_197S_D
NW7_17C_D
Weser6_D
Androscoggin_D
B
Potri.008G092700
Potri.008G175900
Potri.005G190800
Potri.012G005900
Potri.010G053400
Potri.005G031300
Potri.T059900
Potri.008G026400
Potri.001G287500
Potri.008G198100
Potri.019G073800
Potri.T116000
Potri.001G119800
Potri.019G093400
Potri.014G149700
Potri.017G041700
Potri.008G029700
Potri.001G107000
Potri.013G094000
Potri.008G131400
Potri.005G014300
Potri.009G034500
Potri.012G104300
Potri.001G280200
Potri.007G142100
Potri.013G095800
Potri.019G130100
Potri.012G090000
Potri.018G130100
Potri.003G138700
Potri.001G017000
Potri.012G070700
Potri.013G115600
Potri.011G081000
Potri.003G146800
Potri.011G123500
Potri.002G186200
Potri.008G027600
Potri.005G114700
Potri.006G091800
Potri.008G176000
Potri.018G014500
Potri.002G260000
Potri.011G025200
Potri.001G074800
Potri.007G015500
Potri.012G086300
Potri.014G081600
Potri.012G140800
Potri.002G109400
Treatment
Control
Weser4_D
Androscoggin_D
NW7_197S_D
Muhle_Larsen_D
Rochester_D
Max1_D
Weser6_D
NW7_17C_D
Muhle_Larsen_C
Weser6_C
NW7_17C_C
Rochester_C
Weser4_C
Androscoggin_C
NW7_197S_C
Max1_C

Fig. S2

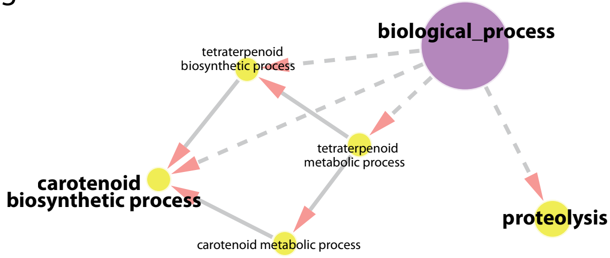
biological_process
tetraterpenoid biosynthetic process
tetraterpenoid metabolic process
carotenoid biosynthetic process
carotenoid metabolic process
proteolysis

Fig. S3

A

	Logo	E-value	Sites	Width	More	Submit/Download
1.		8.7e-010	4207	6		
2.		1.6e-013	2084	6		
3.		3.0e+000	54	6		
4.		2.4e+000	117	6		
5.		2.9e+000	67	9		
6.		2.9e+000	15	6		
7.		3.0e+000	2264	6		
8.		2.3e+000	466	10		
9.		2.2e+000	1736	6		
10.		3.0e+000	10	7		

B

	Logo	E-value	Sites	Width	More	Submit/Download
1.		2.3e-006	3805	6		
2.		3.0e+000	3720	7		
3.		7.6e-009	1411	6		
4.		2.4e+000	2	10		
5.		2.6e+000	31	10		
6.		2.0e+000	4	9		
7.		2.8e+000	7	9		
8.		3.0e+000	2148	6		
9.		3.0e+000	411	8		
10.		3.0e+000	12	6		

C

	Logo	E-value	Sites	Width	More	Submit/Download
1.		3.1e-007	3348	6		
2.		6.7e-008	790	6		
3.		2.4e+000	10	9		
4.		4.2e-001	12	9		
5.		3.0e+000	18	9		
6.		3.0e+000	3723	7		
7.		3.0e+000	2	8		
8.		2.9e+000	9	10		
9.		2.9e+000	3734	6		
10.		2.6e+000	178	6		

Fig. S4

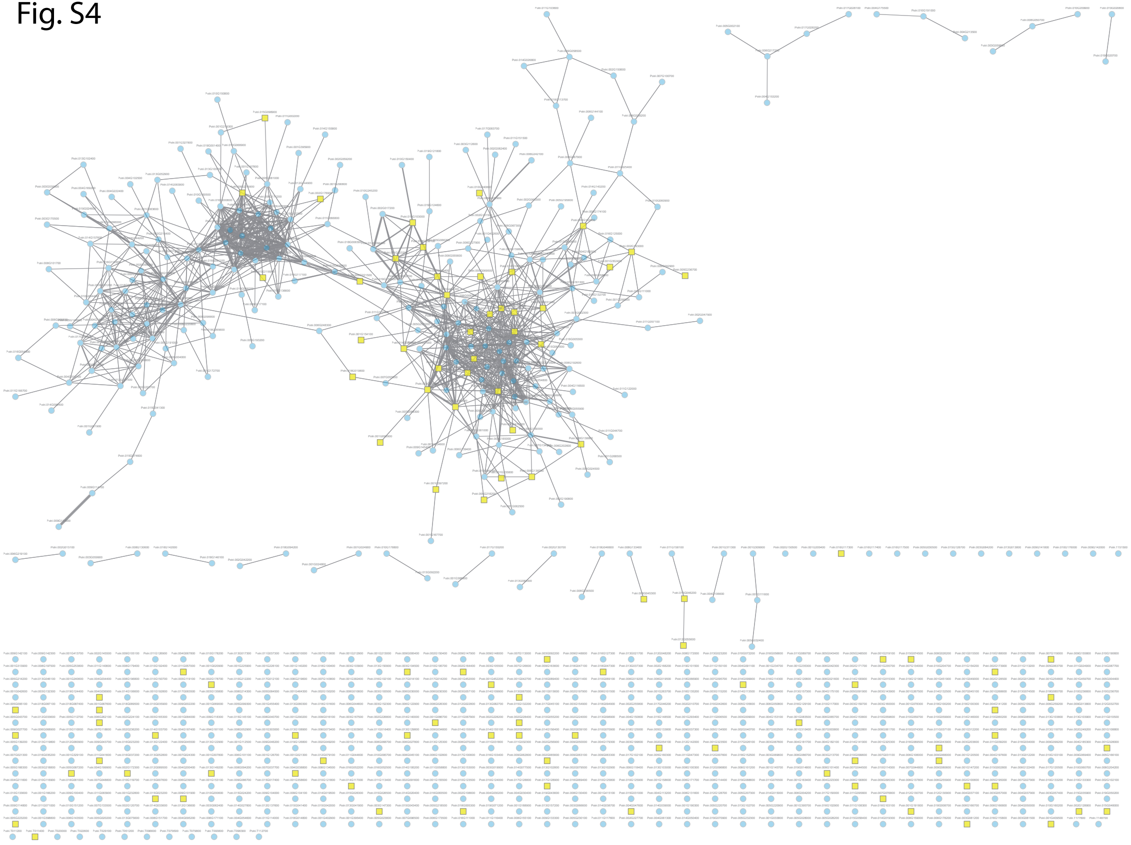

Supplement: Supplementary file 2 — Figure S1. Heatmaps presenting scaled rpkm-values of the top 50 up- and down-regulated genes. Plots were produced with the heatmap() fuction in R with default settings. Pure P. trichocarpa samples are indicated by blue font. Figure S2. GO term analysis of down-regulated genes highlighted the biological processes ‘proteolysis’ and ‘carotenoid biosynthetic process’. Figure S3. Differential motif enrichment analysis by MEME consistently identified the G-box (CACGTG) as the most significant motif. Promoters (1 kb sequence upstream from the transcriptional start site) of the 885 herbivore-induced genes were compared to those of three randomly sampled sets (A to C) of non-induced genes. Figure S4. Co-expression network analysis revealed a network comprising 258 out of the 885 herbivore-induced genes. The network forms two expression modules. Co-expressed genes are connected by lines, whose widths correspond to the strength of the interaction. Transcription factors are depicted in yellow. (PDF 3830 kb) [file 12864_2019_6048_MOESM2_ESM.pdf]
